# A Retrospective Analysis of Colorectal Serrated Lesions from 2005 to 2014 in a Single Center: Importance of the Establishment of Diagnostic Patterns

**DOI:** 10.1155/2018/5946057

**Published:** 2018-10-21

**Authors:** Priscilla S. P. Oliveira, Rita B. Carvalho, Daniela O. Magro, Michel G. Camargo, Carlos A. R. Martinez, Claudio S. R. Coy

**Affiliations:** ^1^Department of Surgery, Medical Sciences School, Campinas State University, Campinas 13083-887, Brazil; ^2^Diagnostic Center of Diseases of the Digestive System-Gastrocentro, Medical Sciences School, Campinas State University, Campinas 13083-878, Brazil

## Abstract

**Background:**

Serrated colorectal lesions are increasingly recognized as an important process in the development of colorectal cancer. Endoscopic and histological diagnosis may be difficult, and knowledge of the serrated lesions is important for the establishment of strategies for treating colorectal lesions. We aimed to analyze serrated lesions diagnosed at a single center and evaluate if there was an increase in their identification over the years.

**Design and Setting:**

A retrospective analysis of colonoscopy reports was performed at a specialized center from 2005 to 2014.

**Methods:**

Colonoscopy reports about any resected endoscopic lesions were reviewed and subjected to histological diagnosis from 2005 to 2014. Then, serrated lesions were evaluated based on morphological characterization, location, size, occurrence of synchronous lesions, and the patient's history of colorectal cancer and polyps.

**Results:**

A total of 2126 colonoscopy examination reports were reviewed, and 3494 lesions were analyzed. On histopathological examination, 1089 (31.2%) were classified as hyperplastic polyps, 22 (0.6%) as sessile serrated adenomas, and 21 (0.6%) as traditional serrated adenomas. There was an increase in the number of cases of sessile and traditional serrated adenomas diagnosed after 2010. Before 2010, two cases of sessile serrated adenomas and seven cases of traditional serrated adenomas were diagnosed; after 2010, 20 cases of sessile serrated adenoma and 14 cases of traditional serrated adenomas were diagnosed.

**Conclusion:**

There was an increase in the diagnosis of sessile serrated adenomas over the years, which can be attributed to better accuracy in colonoscopy and histological classification.

## 1. Introduction

Nowadays, owing to the high incidence of and mortality due to colorectal cancer, there is a need for effective prevention strategies. Since fecal occult blood tests are employed for population screening, colonoscopy is regarded as a gold standard procedure owing to its high sensitivity for detecting polyps. Removal of polyps is associated with a decrease in colorectal cancer incidence, and better knowledge of the molecular colorectal cancer-related pathways involved in precancerous lesions is essential to increase their detection as well as to establish better screening programs.

Until 1990, three types of lesions were associated with the development of colorectal neoplasia: tubular adenomas, tubulovillous adenomas, and villous adenomas. They originate from serial gene mutations, particularly the *APC* and *KRAS* genes, and are known as the classical adenoma-to-carcinoma sequence.

Hyperplastic polyps, which are common in the colon and particularly in the rectum, were considered nonneoplastic lesions for several years [1–3]. In 1990, a group of pathologists identified architectural changes similar to those in hyperplastic lesions in a few colorectal adenomas. These were termed as serrated adenomas [4]. Because of their similar architectural appearance and the suggestion that some of these adenomas may develop from initial hyperplastic polyps, a new group of lesions was defined. These lesions display similar histological characteristics and present diagnostic difficulties to pathologists; therefore, establishment of reproducible criteria for their diagnosis was necessary. The classification of the serrated lesions has been modified over the years since 1990 because knowledge regarding histology has increased. The changes in their histological classification brought difficulties in their correct diagnosis. Only in 2010, the World Health Organization published the latest classification where the serrated lesions were differentiated into sessile serrated adenomas or sessile serrated polyps, traditional serrated adenomas, and hyperplastic polyps. This is the classification of choice for these lesions since then [5–8].

Serrated lesions started receiving increased attention after studies demonstrated that they were more frequent in the right colon as well as their association with faster development into carcinoma, when compared to the classical adenoma-to-carcinoma sequence. The epigenetic origin of cancer cells is where alterations result from the hypermethylation of cytosine- and guanine-rich regions, in which a sequence of events may culminate in the inactivation of the *hMLH1* gene. This pathway is associated with the *BRAF* gene mutation, which is mutually exclusive from the *KRAS* gene mutation, which is involved in the so-called “serrated pathway.” It is considered the second most common mechanism for the development of colorectal cancer [5, 7–12]. On the other hand, traditional serrated adenomas are now known to be secondary to the *KRAS* gene mutation and can evolve through the classical adenoma-to-carcinoma transition [5–7]. So, traditional serrated adenomas mostly resemble conventional adenomas with respect to the endoscopic appearance and molecular behavior.

Nowadays, there are two aspects that may be related to the lower occurrence of the diagnosis of the serrated lesions, ranging from 0.1% to 14.7%: endoscopic detection and histological criteria for the correct diagnosis and classification [13, 14]. In the Brazilian population, there are no studies that evaluate its prevalence and clinical and endoscopic aspects.

We aimed to evaluate the number of serrated lesions diagnosed before and after 2010, when the current histological classification was developed and established, as well as evaluate its localization, size, and morphological features.

## 2. Methods

### 2.1. Study Design

We retrospectively analyzed data from colonoscopies performed at the Gastrocentro of the State University of Campinas (UNICAMP) between January 1, 2005, and December 31, 2014, to determine the number of the serrated lesions diagnosed in this period and if there was an increase in its diagnosis after an increase in the knowledge about this lesion. We also aimed to describe the characteristics of the serrated lesions (morphological characterization, size, and localization) and prevalence of these kinds of lesions regarding all the lesions associated with the development of colorectal neoplasia. Colonoscopies with a positive result for elevated polyps or flat lesions were included. The locations of the lesions were classified as follows: proximal colon (from the cecum to the splenic flexure) and distal colon and rectum (from the splenic flexure to the rectum). Regarding size, the lesion sizes were classified as <10 mm, 10–20 mm, and >20 mm.

The lesions were grouped as conventional adenomas (tubular adenomas, tubulovillous adenomas, and villous adenomas) and serrated lesions (hyperplastic polyps, sessile serrated adenomas, and traditional serrated adenomas). Histological diagnoses were done by different pathologists, and there was no histological review. Usage of histological classification for serrated lesions began in 2005, and the classification has changed over the years. After 2010, the criteria used were those defined by the World Health Organization guidelines, which were issued in that year; these are the same that have been used until today [5].

All colonoscopies were done in the same endoscopy unit but with different endoscopists, and histological diagnoses were analyzed by the Department of Pathology, UNICAMP, using different pathologists but all with experience in the gastrointestinal tract.

### 2.2. Participants

All colonoscopy reports from patients older than 18 years who had undergone resection of at least one lesion and histological analysis were included. The participants were referred to Gastrocentro (a specialized public unit for gastrointestinal diseases of UNICAMP) from the outpatient units of Hospital de Clinicas-UNICAMP with any indication for colonoscopy. Patients with familial adenomatous polyposis, Lynch syndrome, or hyperplastic polyposis syndrome, with imprecise endoscopic descriptions were excluded. Cases with posterior histological diagnosis revision were also excluded.

### 2.3. Variables

Indication for colonoscopy, number of lesions per examination, and aspects related to each resected lesion (size, location, and endoscopic appearance) as well as epidemiological criteria (sex and age) were analyzed. The locations of the lesions were classified as follows: proximal colon and distal colon and rectum. Regarding size, the lesions sizes were classified as <10 mm, 10–20 mm, and >20 mm. The Paris Classification was applied for morphological description [15, 16]. The lesions were grouped into conventional adenomas and serrated lesions.

The associations of different types of serrated lesions with conventional adenomas, as well as with other serrated lesion types, were analyzed.

### 2.4. Data Analysis

The Statistical Package for the Social Sciences (SPSS) software, version 16, (Statistical Package for the Sciences - SPSS Inc., Chicago, IL, USA) was used for descriptive and statistical analyses. The chi-square statistical test was employed, and statistical significance of 5% was considered.

### 2.5. Ethics

The study protocol was approved by the ethics committee of UNICAMP (number 36002414.0.0000.5404).

## 3. Results

### 3.1. Overall Population

Reports of a total of 2126 examinations involving 1772 patients (mean age 61.3 ± 11.5 years) were analyzed. The total number of lesions detected was 3494, from which 1132 (32.4%) were serrated. From 2005 to 2009, 745 examinations with positive results for polyps were performed, and 1381 were performed in the subsequent years (*P* < 0.001). The average number of lesions identified through colonoscopy was 1.65 (1–13), and 761 (35.8%) patients had more than one lesion.

The most common indications for examinations included a history of colorectal cancer and a previous personal history of polyps in the colon and/or rectum (15.9% and 15.1%, respectively). Other indications were a family history of colorectal cancer (2.3%), preoperative evaluation of colorectal cancer (2.1%), unknown (1.6%), and other reasons (64.2%).

The histological type, total number of each lesions, and percentage of the lesion types found via colonoscopies are demonstrated in Table 1.

### 3.2. Sex and Age

Regarding the diagnosis of serrated lesions, there were no differences between sex and mean age. One woman was found to have a sessile serrated adenoma and a traditional serrated adenoma via the same examination.

The mean age of the patients with sessile serrated adenomas was 62 ± 12.1 years, while that of the patients with traditional serrated adenomas was 60.2 ± 16.6 years.

### 3.3. Colonoscopy Indication

In eight patients (four with sessile serrated adenomas and four with traditional serrated adenomas), the indication for colorectal cancer was postoperative cancer follow-up findings. Two patients with sessile serrated adenomas had synchronous colorectal adenocarcinoma detected via preoperative colonoscopy. A history of colorectal polyps was observed in two examinations involving sessile serrated adenomas and in six involving traditional serrated adenomas. No patient with a family history of colorectal cancer had lesions with a histological diagnosis of sessile serrated adenoma or traditional serrated adenoma.

### 3.4. Location, Size, and Morphological Aspects

Most of the sessile serrated adenomas were observed in the proximal colon (*n* = 21, 95.4%), while traditional serrated adenomas were mainly observed in the distal colon and rectum (*n* = 13, 61.9%). One case with a traditional serrated adenoma did not have a descriptive location. Hyperplastic polyps were reported in all segments, with predominance in the distal colon and rectum (*n* = 569, 52.2%), followed by the proximal colon (*n* = 208, 19.1%). The distributions of sessile serrated adenoma and traditional serrated adenoma classified according to size, localization, and morphology are shown in Table 2.

We noted the presence of 15 polyps diagnosed as hyperplastic polyps > 10 mm in the right colon and 6 polyps in the transverse colon.

### 3.5. Association with Conventional Adenomas

Tubular adenomas were detected in 11 examinations involving sessile serrated adenomas and in six involving traditional serrated adenomas, while tubulovillous adenoma was only detected in one examination involving a sessile serrated adenoma. One patient presented with sessile serrated adenoma, traditional serrated adenoma, and synchronous tubular adenoma.

### 3.6. Synchronous Serrated Lesions

Five examinations involving hyperplastic polyps showed concomitant sessile serrated adenomas, and four showed concomitant traditional serrated adenomas. One patient presented with three traditional serrated adenomas in the same examination, and two other patients demonstrated more than one serrated lesion in the same examination (one with two traditional serrated adenomas and the other with one traditional serrated adenoma and one sessile serrated adenoma). An association with cancer was observed in two examinations involving sessile serrated adenomas and in one involving traditional serrated adenoma.

### 3.7. Absolute Value of Sessile Serrated Adenomas and Traditional Serrated Adenomas

**Figure 1:**
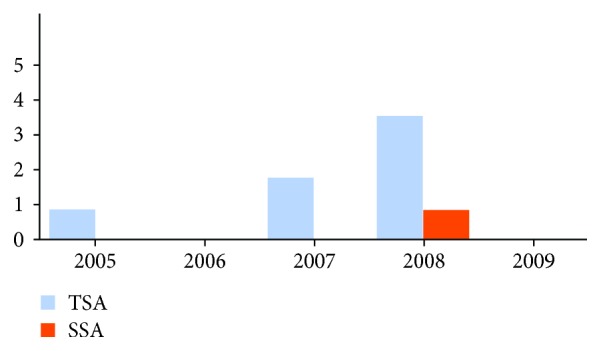
Number of sessile serrated adenomas and traditional serrated adenomas from 2005 to 2009 (year; lesions diagnosed—absolute value). One case of traditional serrated adenoma was diagnosed in 2005, two in 2007, and four in 2008. One case of sessile serrated adenoma was diagnosed in 2008.

**Figure 2:**
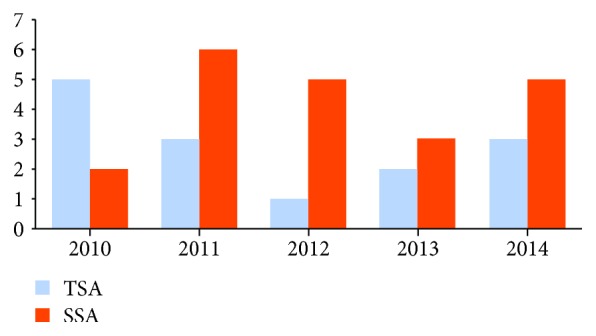
Number of sessile serrated adenomas and traditional serrated adenomas from 2010 to 2014 (year; lesions diagnosed—absolute value). Two sessile serrated adenomas were diagnosed in 2010, six in 2011, five in 2012, three in 2013, and five in 2014. The number of traditional adenomas diagnosed was five in 2010, three in 2011, one in 2012, two in 2013, and three in 2014.

The number of sessile serrated adenomas and traditional serrated adenomas showed a large variation over the study period, with an increase in the last 5 years (Figures 1 and 2).

No case of sessile serrated adenoma or traditional serrated adenoma was diagnosed in 2006 and 2009. After 2010, there was an increase in the number of serrated lesions diagnosed, with an average of 7 lesions per year. Two sessile serrated adenomas and seven traditional serrated adenomas were diagnosed between 2005 and 2009, while 20 sessile serrated adenomas and 14 traditional serrated adenomas were diagnosed between 2010 and 2015, showing a significant increase in the recent years (*P* < 0.001). The relative frequencies of traditional serrated adenomas and sessile serrated adenomas before and after 2010 were 0.8% and 2.3%, respectively.

## 4. Discussion

The diagnosis of serrated lesions may be challenging. For many years, serrated lesions were all classified as hyperplastic and nonneoplastic lesions. Today, owing to better knowledge about molecular pathways, the serrated lesions are known to be a more aggressive form of precancerous lesions. Therefore, accurate identification and diagnosis have practical implications in screening for colorectal cancer, and the analysis of a case by case may contribute to better accuracy.

When comparing serrated lesions with other adenomas, the prevalence of hyperplastic polyps in this sample was as expected (i.e., one-third of all polyps). However, when analyzed in comparison with serrated lesions only, hyperplastic polyps were the majority (96.2%). This value was higher than that in the literature, although other studies also found hyperplastic polyps to be the most frequent type (75% of all serrated lesions) [5, 12, 13].

However, the discrepancy in the prevalence of sessile serrated adenomas was high. This sample in this study showed a prevalence of 0.6%, which was close to the minimum values (0.1% to 14.7%), relative to all polyps found in the colon. However, when only serrated lesions were analyzed (1.9%), the prevalence was lower (they are generally estimated to comprise up to 25% of all serrated lesions) [5, 7, 17]. The prevalence of traditional serrated adenomas, which is considered rare, was within the expected range [5, 14, 18].

This large difference in the prevalence of hyperplastic polyps and sessile serrated adenomas between this study and other reports may be explained by the different classifications adopted since the lesions were first discovered. Moreover, studies that evaluated the prevalence of sessile serrated adenomas reported a higher prevalence of sessile serrated adenoma lesions based on histological reviews [4, 6, 9, 13, 18, 19].

Furthermore, the difference between the criteria for the diagnosis of sessile serrated adenomas may have been a confounding factor that affected the different prevalence rates in different studies as more knowledge has been acquired over the years [5, 15, 19, 20].

Since histological aspects of sessile serrated adenomas can cause them to be mistaken for hyperplastic polyps, this may also justify the low prevalence of sessile serrated adenomas compared with hyperplastic polyps. Moreover, the difficulty in the diagnosis of superficial elevated lesions through colonoscopies owing to their endoscopic characteristics may also have contributed to their lower prevalence.

The retrospective design of the study also contributed to this difference. Although the histological and endoscopic examinations were performed at a referral center for gastrointestinal tract pathologies, the examiners could not be selected.

Although there were divergences among published data on the prevalence of sessile serrated adenomas, the same was not true for their location. Sessile serrated adenomas were often observed in the proximal colon, and their appearance (sessile and/or elevated) matched that in the literature. As in other studies, no sessile serrated adenoma was diagnosed in the distal colon and rectum [9, 21]. Traditional serrated adenomas were present in greater numbers in the distal colon and rectum, as was expected.

Regarding the endoscopic aspects of the Paris Classification, there was a predominance of sessile lesions, with only one elevated lesion. This supports the hypothesis that lesions may have gone unnoticed during colonoscopies, contributing to their low prevalence. Endoscopic identification of sessile serrated adenomas is difficult. The lesions are flat or slightly elevated, with no substantial changes in color, and they have imprecise limits and are covered by mucus that is difficult to remove [5, 7, 12]. A better visualization of these lesions can be achieved with high-definition devices and chromoscopy [9, 17, 22, 23].

Another important examination-related aspect that may have contributed to the low prevalence of sessile serrated adenomas is that colorants were not routinely used in the right colon. Despite the importance of their role in the diagnosis of sessile serrated adenomas, there have been no reports on their use in examinations that detected sessile serrated adenomas or hyperplastic polyps > 10 mm in the proximal colon [12, 22].

The difference between the prevalence of serrated lesions in this study and in the published data, as well as the differences in the distribution and macroscopic characteristics, raises questions about the quality of diagnoses and whether there have been any improvements from the increased experience of endoscopic examiners and pathologists. Payne et al. [24] reported an association between the rate of resection of adenomas and the diagnosis of serrated lesions. Therefore, endoscopic examinations performed by more experienced endoscopists will yield a high detection of serrated lesions.

We observed an increase in the number of sessile serrated adenomas and traditional serrated adenomas diagnosed within the last 5 years of the study period. In the last 5 years, there were no changes in the histological classification of the serrated lesions, and the pathologists have more years of experience with these kind of lesions.

Data for this study were collected from one of the region's reference centers for the treatment of colorectal cancer and inflammatory bowel disease. Therefore, most of the patients submitted to colonoscopies presented risk factors for the development of precursor lesions or colorectal cancer, as evidenced by the indications for the examinations. On the other hand, previous data were mostly related to screening tests that were performed in asymptomatic patients [25–28].

A relevant aspect of this study is regarding the occurrence of synchronous lesions. The simultaneous occurrence of conventional adenomas and sessile serrated adenomas or traditional serrated adenomas was 51.28%. Previous reports showed that the presence of conventional adenomas with serrated lesions was uncommon [28].

The prevalence of serrated lesions in this study differed from that in other studies in some ways, since we detected a higher number of hyperplastic polyps and observed very similar distributions between sessile serrated adenomas and traditional serrated adenomas. The increases in the number of examinations performed and in the number of serrated lesions diagnosed were significant; there was an increase in the relative frequency of serrated lesions. Therefore, an increase in the diagnosis of serrated lesions was observed after 2010.

However, this study has some limitations. Our study is a retrospective analysis that was conducted at a single center, but histological reports as well as endoscopic examinations were done by various pathologists and endoscopists, certainly with heterogeneous descriptions. For this reason, it was difficult to state the number of colonoscopies without lesions. In recent years, the awareness regarding endoscopic appearance on serrated lesions particularly in the right colon contributed to better accuracy. Unfortunately, the use of chromoendoscopy or magnification techniques like narrow band imaging was not always employed in the unit of date origin of this study because the heterogeneity of the colonoscopists. Besides that, before 2010, many lesions that are now classified as sessile serrated adenomas were classified as hyperplastic (before the adoption of the criteria for histological classification published by the World Health Organization for serrated lesions in 2010). Hence, we did not review the histological features, which could have influenced the number of the serrated lesions detected.

## 5. Conclusion

There was an increase in the diagnosis of sessile serrated adenomas after 2010, which can be attributed to better accuracy of colonoscopy and the histological classification.

## Figures and Tables

**Table 1:** Histological type, total number of each lesion (frequency), and percentage of the lesions found via colonoscopies along with distribution of all the histological types of only serrated lesions.

	*n*	%
*Histological type*		

Tubular adenoma	2085	59.7
Hyperplastic polyp	1089	31.2
Tubulovillous adenoma	217	6.2
Adenocarcinoma	46	1.3
Sessile serrated adenoma	22	0.6
Traditional serrated adenoma	21	0.6
Villous adenoma	14	0.4
Total	3494	100

*Serrated lesions*		

Hyperplastic polyp	1089	96.2
Sessile serrated adenoma	22	1.94
Traditional serrated adenoma	21	1.86
Total	1132	100

**Table 2:** Descriptive analysis of serrated lesions. The distribution of the serrated lesions according to their general aspects: the mean age, localization, and size and morphological characteristics.

		Sessile serrated adenoma (SSA)	Traditional serrated adenoma (TSA)
Mean age		62 ± 12.1	60.2 ± 16.6

Location	Proximal colon	21–95.4%	7–33.3%
	Distal colon and rectum	1–4.6%	13–61.9%
	No description	—	1–4.8%

Size	<10 mm	15–68.2%	12–57.1%
	10–20 mm	7–31.8%	5–23.8%
	>20 mm	—	1–4.8%
	No description	—	3–14.3%

Morphology	Sessile	13–59.1%	17–80.9%
	Superficially elevated	9–40.9%	1–4.8%
	Pedunculated	—	2–9.5%
	No description	—	1–4.8%

## Data Availability

The data used to support the findings of this study are available from the corresponding author upon request.
